# Precipitous Delivery Masquerading as Biliary Colic in the Setting of Depo-Provera® Failure

**DOI:** 10.7759/cureus.6776

**Published:** 2020-01-26

**Authors:** Allen D Chang, Grant S Lipman

**Affiliations:** 1 Emergency Medicine, Stanford University Hospital, Palo Alto, USA

**Keywords:** contraception failure, biliary colic, precipitous delivery, prenatal care, unknown pregnancy, academic emergency medicine, depo provera failure, emergency obstetrics, footling breech, long-acting reversible contraception

## Abstract

Precipitous delivery in the emergency department is a high-acuity, low-occurrence event that requires rapid recognition and interdepartment cooperation to prevent fetal and maternal morbidity and mortality. Prompt recognition of the peripartum state can be delayed by reported usage of long-acting contraception and concurrent distracting complaints. In this case report, a young female presented to the emergency department with epigastric abdominal pain in the setting of recent workup for biliary colic and multiple doses of long-acting, depot contraceptive agents. Early utilization of bedside ultrasound confirmed a full-term, intrauterine pregnancy as well as an impacted gallbladder stone, followed by a precipitous footling breech presentation that required an emergent cesarean section.

## Introduction

Emergency physician assessment of young, sexually active female patients who present with abdominal complaints should always consider pregnancy-related medical conditions. Regardless of self-reported compliance with various contraception methods, formal lab screening for pregnancy should still be completed, recognizing the inherent treatment failure rates of common forms of contraception. Bedside ultrasound can also be useful in rapid evaluation when the patient history is inconclusive, especially in the setting of confounding, multiple complaints that may distract the emergency physician from pregnancy-related conditions.

## Case presentation

A 28-year-old female presented to the emergency department (ED) with crampy, epigastric abdominal pain which had worsened over several hours. This pain was intermittent over the past six months, and was associated with food intake and Depo-Provera® (depot medroxyprogesterone acetate [DMPA]) injections, most recently administered one month prior to this visit. She was scheduled for an outpatient ultrasound for biliary colic the following week. Immediately prior to ED evaluation, she reported clear yellow vaginal discharge that she suspected was urine.

The patient’s vital signs were normal, and her exam demonstrated epigastric and right upper quadrant tenderness to palpation, an obese body habitus, and a normal external genital exam. Serum pregnancy test was positive, and liver function tests included a total bilirubin of 0.3 (normal < 1.2 mg/dL), aspartate aminotransferase 16 (normal 10-35 U/L), alanine aminotransferase 11 (normal 10-35 U/L), alkaline phosphatase 190 (normal 35-105 U/L), and lipase 144 (normal 13-60 U/L). All other lab tests were unremarkable and within normal limits.

**Figure 1:**
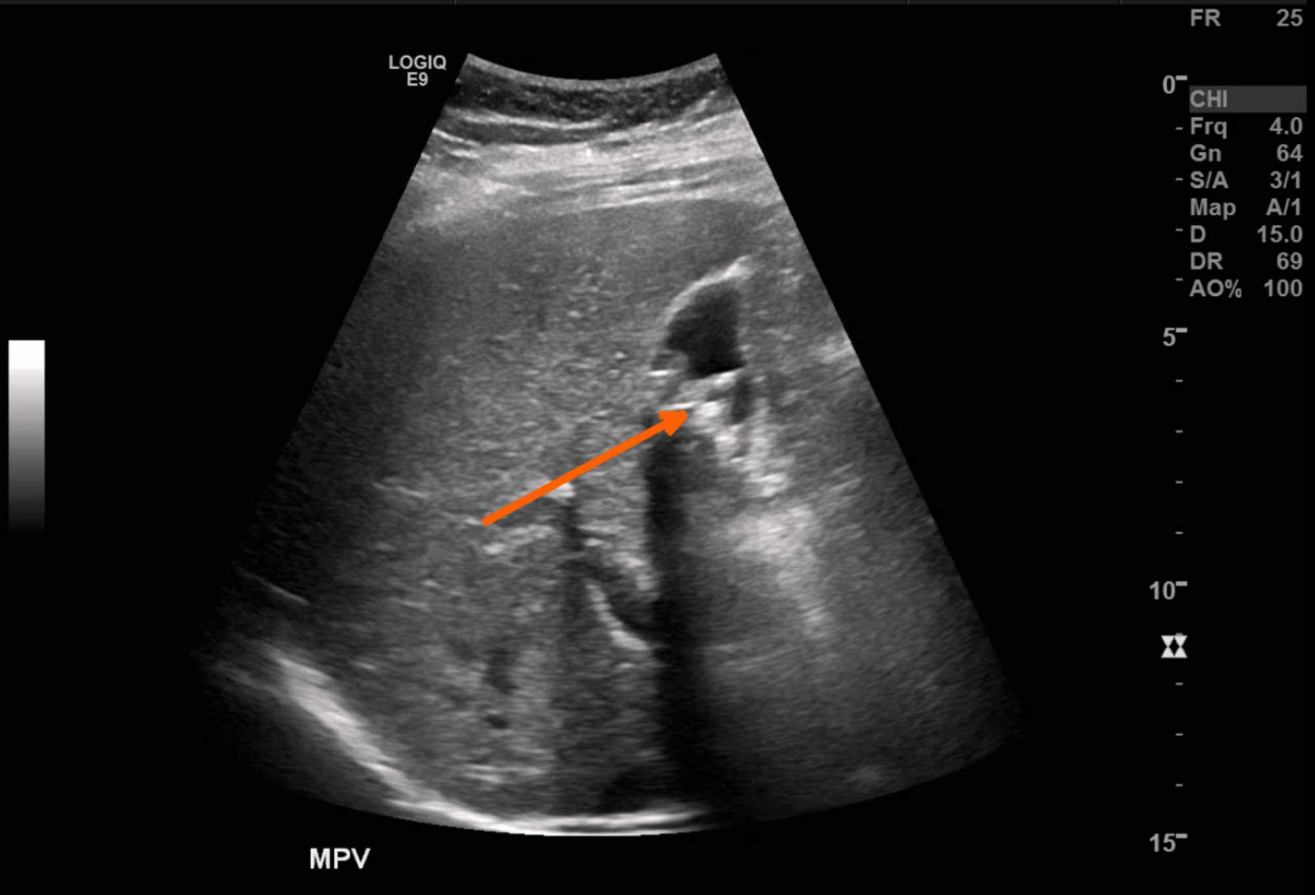
Abdominal ultrasound of the right upper quadrant No pericystic fluid or gallbladder wall thickening. The arrow pointing to impacted stone in gallbladder neck.

ED bedside ultrasound was utilized, demonstrating large, non-mobile gallstones, including one impacted in the gallbladder neck (Figure 1). An adjacent, mature calvarium in a non-vertex position with a normal fetal heart rate was also visualized.

Due to the patient’s persistent tenderness while denying contractions or other signs of active labor, a formal ultrasound was ordered to assist with surgical evaluation of her cholelithiasis. Shortly after, the ED physician team was called to the ultrasound suite where the patient reported feeling movement in her vagina. Upon arrival, a neonatal foot was protruding from the vaginal introitus, consistent with a footling breech presentation. The patient was instructed not to push, and both obstetric and neonatal intensive care unit assets were mobilized to the ED.

The patient was given 2 g of magnesium intravenously by obstetrics and gynecology, and consented to an emergent cesarean section due to the footling breech presentation in the setting of high-risk pregnancy without prenatal care. In the operating suite, a full-term, 7.12 lb baby girl was delivered with Apgar scores of 4 and 8, and was subsequently discharged from an uneventful stay in the neonatal intensive care unit. Our patient eventually had an uneventful laparoscopic cholecystectomy 15 months later.

## Discussion

The most common form of birth control used in the United States is the oral contraceptive pill, with annual failure rates estimated at approximately 9% for the general population. In contrast, long-acting reversible contraceptive methods, including intrauterine devices and subdermal implants, have very low failure rates (<1%), are not user-dependent, and can safely last upwards of 5-10 years [1].

In terms of longevity, between the multi-year efficacy of implantable methods and the short duration of oral pills is DMPA injection, commonly known as Depo-Provera, a long-acting injectable contraceptive with a three-month window of effectiveness. Depot contraception has been found to have superior efficacy to the pill, patch, and ring methods, with rates of failure unaltered by a woman’s age [2]. Approximately 23% of sexually active women in the United States in the mid-to-late 2000s reported using Depo-Provera [3]. Treatment failure in this group is most often related to delayed injections (>90 days from prior administration) or incorrect intramuscular medication placement [4,5].

Our patient completed her most recent depot injection one month prior to her ED evaluation. Given her full-term/third-trimester presentation, we later calculated that she had received a total of three injections during the course of this pregnancy. It was unclear whether her outpatient clinic tested her for pregnancy prior to administration, as she received her injections within the 90-day recommended interval as scheduled.

In one review, among unplanned pregnancies in women using Depo-Provera, 46% were diagnosed after the first trimester, and 19.1% received additional injections while pregnant. Among this cohort, authors considered that some of these pregnancies predated Depo-Provera use, which more accurately represents a failure to recognize pregnancy before administration rather than failure of treatment itself [5].

Given the low rate of this type of contraceptive failure, pregnancy screening of asymptomatic women at the time of re-injection is not routinely recommended for providers [5]. In our patient, her unawareness and lack of classic pregnancy symptoms unfortunately prevented her from seeking prenatal evaluation and care. Of note, denial of pregnancy is an important condition that is more common than expected, with an incidence at 20 weeks' gestation of approximately one in 475 [6]. A study in 2011 found that, contrary to prior belief, women who deny the veracity of their pregnancy are a much more heterogeneous group without clear-cut identifying or unifying factors [7]. As a result, calculation of risk scores and stratification is challenging, and physicians are reminded to be continuously observant for signs and symptoms of pregnancy in women of childbearing age.

Regardless of self-reported contraceptive history, emergency medicine evaluation of young females presenting with abdominal pain should include testing for pregnancy, given the potential for contraceptive failure and inconsistent reporting of sexual activity. Emergency physicians are encouraged to periodically review obstetric/precipitous delivery equipment, interdepartment protocol, and nursing training in order to optimize coordination of resources in this time critical presentation [8].

Concurrent biliary colic and a full-term, precipitous delivery in the setting of injectable hormonal contraception failure is a unique combination. In this case, high clinical suspicion, point-of-care lab testing, and bedside ultrasound expedited the management of this dual diagnosis toward an overall positive outcome.

## Conclusions

With an annual incidence of <1%, failure of depot hormonal contraception is a rare but known entity, even in the setting of medication compliance and serial administrations. Regardless of patient-reported compliance with medication, emergency physicians should still screen for pregnancy in young women with abdominal pain. Rapid recognition of precipitous delivery in the emergency department can be facilitated by early utilization of ultrasound and prompt mobilization of additional medical resources.
